# Characterization of the Dynamic Changes of Ruminal Microbiota Colonizing Citrus Pomace Waste during Rumen Incubation for Volatile Fatty Acid Production

**DOI:** 10.1128/spectrum.03517-22

**Published:** 2023-03-02

**Authors:** Shiqiang Yu, Liuxue Li, Huiying Zhao, Yan Tu, Ming Liu, Linshu Jiang, Yuchao Zhao

**Affiliations:** a Beijing Key Laboratory of Dairy Cow Nutrition, Animal Science and Technology College, Beijing University of Agriculture, Beijing, China; b Beijing Key Laboratory of Dairy Cow Nutrition, Key Laboratory of Feed Biotechnology of the Ministry of Agriculture and Rural Affairs, Institute of Feed Research, Chinese Academy of Agricultural Sciences, Beijing, China; c Beijing Beinong Enterprise Management Co., Ltd., Beijing, China; Royal Agricultural University

**Keywords:** anaerobic digestion, citrus pomace, fermentation profile, microbial community, rumen fluid, citrus waste utilization, rumen fermentation

## Abstract

Rumen microorganisms are promising for efficient bioconversion of lignocellulosic wastes to biofuels and industrially relevant products. Investigating the dynamic changes of the rumen microbial community colonizing citrus pomace (CtP) will advance our understanding of the utilization of citrus processing waste by rumen fluid. Citrus pomace in nylon bags was incubated in the rumen of three ruminally cannulated Holstein cows for 1, 2, 4, 8, 12, 24, and 48 h. Results showed that total volatile fatty acids concentrations and proportions of valerate and isovalerate were increased over time during the first 12 h. Three major cellulose enzymes attached to CtP rose initially and then decreased during the 48-h incubation. Primary colonization happened during the initial hours of CtP incubation, and microbes compete to attach CtP for degrading easily digestible components and/or utilizing the waste. The 16S rRNA gene sequencing data revealed the diversity and structure of microbiota adhered to CtP were distinctly different at each time point. The increased abundance of Fibrobacterota, *Rikenellaceae_RC9_gut_group*, and *Butyrivibrio* may explain the elevated volatile fatty acids concentrations. This study highlighted key metabolically active microbial taxa colonizing citrus pomace in a 48-h *in situ* rumen incubation, which could have implications for promoting the biotechnological process of CtP.

**IMPORTANCE** As a natural fermentation system, the rumen ecosystem of ruminants can efficiently degrade plant cellulose, indicating that the rumen microbiome offers an opportunity for anaerobic digestion to utilize biomass wastes containing cellulose. Knowledge of the response of the *in situ* microbial community to citrus pomace during anaerobic fermentation will help improve the current understanding of citrus biomass waste utilization. Our results demonstrated that a highly diverse rumen bacterial community colonized citrus pomace rapidly and continuously changed during a 48-h incubation period. These findings may provide a deep understanding of constructing, manipulating, and enriching rumen microorganisms to improve the anaerobic fermentation efficiency of citrus pomace.

## INTRODUCTION

Global population growth enhanced the demand for food processing and heightened waste production concomitantly. Therefore, the utilization of food waste biomass resources has received increasing attention. Anaerobic digestion of food waste has been widely studied to improve the digestion performance of biogas production and solids reduction (1, 2). During anaerobic digestion, a complex anaerobic microbiome transforms organic wastes into volatile fatty acids (VFA) and biogas, a renewable energy source (3). *Citrus* is one of the most important and widely consumed fruit crops with high economic and medicinal value. A huge quantity of citrus wastes and by-products are annually generated by fruits processing industries. Citrus pomace (CtP), the main waste obtained from the industrial production of juice and essential oils, mainly contains large amounts of pectin, cellulose, hemicellulose, and simple sugars (4). However, most CtP is disposed of as waste without being recycled or processed into value-added products (5). Therefore, anaerobic digestion of CtP is needed to utilize the wastes for bioenergy production and minimize the adverse impact of waste disposal on the environment. However, the recalcitrant structure consisting of cellulose encapsulated in a hemicellulose-pectin-lignin matrix remains a major challenge hindering the utilization of cellulosic biomass (6).

Ruminants and rumen microbiota, the community of microorganisms inhabiting the rumen, have evolved in close association with each other for millions of years. The rumen microbiome is usually considered one of the most complex and efficient anaerobic microbial ecosystems in the fermentation of plant biomass (7). Rumen microorganisms have been used for biofuel production from various lignocellulosic wastes such as corn straw (8), rich straw (9), corn stover (10), and wheat straw (11). Various strategies such as pretreatment, bioaugmentation, and employment of enriched rumen microbiota have been used to treat these lignocellulosic wastes for effective liquid and gaseous fuel production (12). It has also been reported that during lignocellulose digestion, rumen microbes colonize cellulose much faster than microbes from other sources, such as leachate (9). Therefore, exploiting ruminal microbes as an efficient inoculum during the anaerobic fermentation of citrus waste biomass is attractive.

The rumen is one of four stomach compartments in ruminants, and rumen fluid hosts a complex anaerobic microbial ecosystem consisting of 10^10 to 11^ cells/mL bacteria, 10^6^cells/mL archaea, 10^3 to 5^ cells/mL fungi, and 10^4 to 6^ cells/mL protozoa (13), which interact in ways that have a significant influence on the utilization of plant materials. Methane production dominated by archaea is an inherent consequence of plant biomass degradation by bacteria in the rumen that allows continuous microbial activity (14). Besides cellulose, citrus biomass contains high contents of plant bioactive compounds such as polyphenols, tannins, and flavonoids (15). Interactions between these compounds and microbes not only have beneficial effects on the host, but can also inhibits methanogens (16), which would affect CtP utilization by rumen microorganisms.

Currently, the available anaerobic digestion reactors are much less effective than animal rumen (17). An in depth understanding of lignocellulose digestion mechanisms in ruminants can provide valuable insights for various biotechnology applications such as the production of industrially relevant enzymes (cellulases, hemicelluloses, ligninases), biofuels (biohydrogen, biogas) and VFA (17). Most studies with rumen fluid as the inoculum focused on the dynamic change of rumen microorganisms associated with the liquid phase in anaerobic digesters during biomass fermentation (8, 18). However, it was reported that liquid-phase bacteria only comprise 20 to 30% of the total microbes (19). Therefore, studying the ruminal solid-phase bacteria during *in situ* fermentation would greatly assist our understanding of dynamic changes of dominant microbes for degrading CtP. In the present study, 16S rRNA sequencing analysis of the microbes adhering to CtP in the rumen of dairy cows was undertaken to identify the key microbes contributing to CtP degradation and VFA production.

## RESULTS AND DISCUSSION

### Fermentation parameters.

The rumen is, in effect, an anaerobic fermenter for cellulose decomposition. The dynamics of fermentation characteristics in the 48-h *in situ* incubation are shown in Table 1. Rumen fluid pH is an important indicator for evaluating the fermentation process. Compared with the value at 1 h, pH was numerically but not significantly reduced in subsequent time points. The lower rumen pH might be usually associated with the higher total VFA concentration (20). The decomposition of lignocellulosic material by rumen microbes results in forming of VFA (21). As expected, total VFA (TVFA) concentration was significantly increased with CtP fermentation (*P* < 0.05), with the greatest values be observed in 8 to 24 h, and there was a significant reduction from 24 to 48 h (*P* < 0.05). The ability to convert plant lignocellulosic material into VFA for rumen's microbiome is very important for the nutrition of the host ruminant, and these metabolites are easily absorbed into the host's bloodstream and assimilated as the primary source of nutrients (21). It is important to realize that ruminal TVFA concentration rarely reflects the amount produced in the rumen since there is a dynamic balance between production and disappearance (22). In the present study, the increased TVFA concentrations indicated that rumen organisms could ferment CtP to lead to VFA accumulation. There were no significant differences in acetate, propionate, butyrate, and isobutyrate proportions (*P* > 0.05) among various time points. However, valerate and isovalerate proportions increased from 1 to 8 h. The branch-chain VFA is required for most fiber-degrading microbes in the rumen (23). Therefore, increased isovalerate can be advantageous for the growth of cellulolytic bacteria.

**TABLE 1:** Changes of ruminal fermentation parameters within a 48-h *in situ* incubation of citrus pomace in the rumen^*a–c*^

Item	Time							SEM	*P* value
	1 h	2 h	4 h	8 h	12 h	24 h	48 h		
pH	6.34	6.09	6.11	6.15	6.14	6.27	6.07	0.272	0.943
TVFA^*d*^, mmol/L	106.37^*c*^	115.62^*b*^	119.42^*ab*^	123.71^*a*^	122.42^*a*^	120.10^*a*^	107.01^*c*^	4.890	0.023
Acetate, %	60.17	60.28	58.62	57.89	59.00	59.29	59.68	1.357	0.945
Propionate, %	21.63	20.92	20.34	20.08	20.28	22.44	22.43	0.989	0.779
Butyrate, %	11.90	11.95	13.87	14.33	12.86	11.62	11.89	1.335	0.314
Isobutyrate, %	2.10	1.94	1.71	1.93	1.87	1.96	1.59	0.780	0.996
Valerate, %	2.14^*c*^	2.54^*abc*^	2.65^*ab*^	2.94^*a*^	2.77^*ab*^	2.36^*bc*^	2.23^*c*^	0.205	0.015
Isovalerate, %	2.05^*c*^	2.36^*b*^	2.80^*a*^	2.83^*a*^	2.93^*a*^	2.34^*b*^	2.17^*bc*^	0.129	<0.001
NH_3_-N^*e*^, mg/dL	13.14	12.78	12.62	11.37	10.89	11.75	12.06	1.148	0.463
Acetate/propionate	2.82	2.93	2.95	2.92	2.89	2.65	2.67	0.380	0.964
MCP^*f*^, mg/mL	1.44	1.53	1.58	1.49	1.46	1.57	1.54	0.106	0.760

a–c Means within a row followed by different lower-case letters differ significantly from each other (*P* < 0.05).

d TVFA, total volatile fatty acids.

e NH_3_-N, ammonia-nitrogen.

f MCP, microbial crude protein.

### Cellulose enzyme activity and biomass degradation.

Cellulosic biomass can be converted into biofuel through enzyme hydrolysis, which can be achieved chemically or biologically. Rumen microbial consortium is a highly effective, coevolved ecosystem with a broad spectrum of enzymes involved in cellulose breakdown. As expected, the activities of the three main cellulose enzymes attached to CtP were generally high at the early fermentation stage and subsequently reduced as the fermentation process progressed (Table 2). The activities of these enzymes significantly decreased after 12 h, indicating that the degradation efficiency was significantly decreased during the late fermentation stage. During the fermentation period, the relative biomass of CtP, including dry matter (DM), neutral detergent fiber (NDF), and acid detergent fiber (ADF), decreased over time. A significant decrease in NDF and ADF biomass were observed after 12 h, and an increase in TVFA accompanied this decrease. Cellulose degradation may enhance the accessibility and digestibility of plant biomass for anaerobic fermentation (24). In the present study, CtP was placed in the rumen for *in situ* fermentation, which made CtP constantly in contact with the metabolically active microbial community. Our results provided an effective reference for the optimal utilization efficiency of CtP biomass via the rumen microorganism assistance.

**TABLE 2:** Changes of attached cellulose enzyme activities and biomass degradation of citrus pomace within a 48-h *in situ* incubation in the rumen^*a–e*^

Item	Time							SEM	*P* value
	1 h	2 h	4 h	8 h	12 h	24 h	48 h		
Cellulose enzyme, U/g									
Pectinase	2.14^*c*^	2.92^*b*^	3.73^*a*^	3.17^*ab*^	3.61^*a*^	2.23^*c*^	2.30^*c*^	0.239	<0.001
Carboxymethyl cellulase	0.55^*e*^	2.29^*a*^	2.04^*b*^	1.60^*d*^	1.93^*c*^	0.61^*e*^	0.54^*e*^	0.389	<0.001
Xylanase	0.43^*d*^	0.64^*cd*^	1.08^*a*^	0.83^*bc*^	0.95^*ab*^	0.39^*d*^	0.47^*cd*^	0.089	<0.001
Relative biomass, %									
DM^*f*^	90.22^*a*^	86.76^*ab*^	82.34^*b*^	77.32^*b*^	65.49^*c*^	46.21^*d*^	28.17^*e*^	4.871	<0.001
NDF^*g*^	95.38^*a*^	92.17^*a*^	87.43^*ab*^	83.87^*ab*^	74.19^*b*^	59.83^*c*^	42.36^*d*^	4.729	<0.001
ADF^*h*^	97.64^*a*^	94.55^*a*^	92.17^*ab*^	86.45^*ab*^	80.12^*b*^	68.56^*c*^	55.68^*d*^	4.011	<0.001

a–e Means within a row followed by different lowercase letters differ significantly from each other (*P* < 0.05).

f DM, dry matter.

g NDF, neutral detergent fiber.

h ADF, acid detergent fiber.

### Relative abundances of specific rumen microbes by real-time qPCR.

The relative abundances of specific rumen microbes were quantified by qPCR. The dynamics of the relative abundances of nine microbes are presented in Table 3. As an essential part of the solid adherent biofilm, methanogens are attracted to feed particles by metabolites from c cellulolytic bacteria (25). With prolonging the fermentation, the abundance of methanogens was reduced continuously. The reason may be that the growth and activity of methanogens was inhibited directly by tannin or polyphenols in CtP (26). *Rumicoccus albus*, Ruminococcus flavefaciens, Butyrivibrio fibrisolvens, and Fibrobacter succinogenes are usually considered main cellulolytic bacteria in the rumen (27). In the present study, the relative abundance of Butyrivibrio fibrisolvens increased first and then decreased, with a maximum value observed at 24 h. The results indicated that Butyrivibrio fibrisolvens was important in degrading CtP biomass. Our analysis of specific rumen microbes using qPCR suggested that dynamic microbiome changes mediate the decomposition of CtP. Therefore, a detailed investigation using high-throughput techniques is required.

**TABLE 3:** Changes in relative abundances (% of total bacteria) of several specific rumen microbes attached to citrus pomace within a 48-h *in situ* incubation in the rumen

Item	Time							SEM	*P* value
	1 h	2 h	4 h	8 h	12 h	24 h	48 h		
Methanogens, ×10^−3^	3.68^*ab*^	3.66^*ab*^	4.28^*a*^	3.18^*b*^	1.50^*c*^	2.26^*c*^	1.66^*c*^	0.384	<0.001
*Anaerovibrio lipolytica*, ×10^−3^	6.51	8.85	8.03	4.39	6.82	3.87	4.52	0.228	0.270
Butyrivibrio fibrisolvens, ×10^−1^	0.58^*b*^	0.54^*b*^	0.87^*ab*^	0.82^*ab*^	1.01^*a*^	1.04^*a*^	0.67^*b*^	0.404	0.022
Fibrobacter succinogenes, ×10^−2^	1.75	1.89	1.82	1.23	2.16	1.79	1.78	0.086	0.648
*Megaspheara elsdenii*, ×10^−3^	1.04	1.26	1.29	0.94	1.16	1.89	1.47	0.138	0.857
Prevotella brevis, ×10^−2^	1.44^*b*^	0.96^*b*^	1.91^*b*^	2.38^*ab*^	2.49^*ab*^	3.38^*a*^	3.78^*a*^	0.069	0.012
*Rumicoccus albus*, ×10^−2^	0.99	1.76	1.54	0.83	1.14	0.92	0.85	0.611	0.645
*Rumicoccus flavefaciens*, ×10^−3^	1.71	2.45	2.38	2.60	2.17	2.12	1.35	1.784	0.992
Streptococcus bovis, ×10^−3^	3.31	4.22	3.23	2.26	2.26	1.64	1.97	1.923	0.833

a–c Means within a row followed by different lowercase letters differ significantly from each other (*P* < 0.05).

### Taxonomic profiling of ruminal bacterial community attached to CtP.

**FIG 1:**
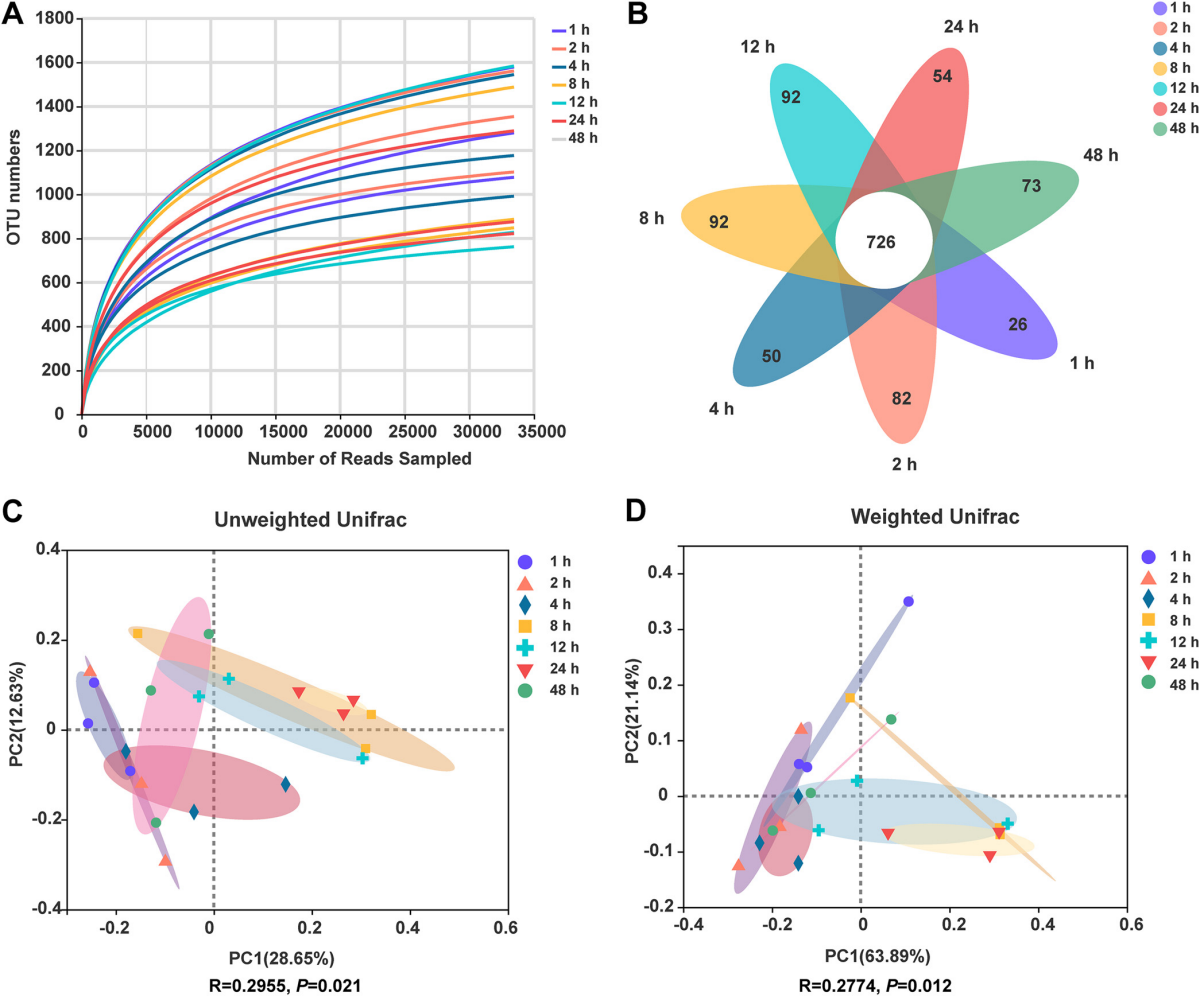
Dynamic changes of the structure of ruminal bacteria colonizing citrus pomace. (A) The rarefaction curves; (B) Venn diagram at OTU level; (C) PCoA plot based on unweighted Unifrac distance; (D) PCoA plot based on weighted Unifrac distance.

In total, the amplicon sequencing of ruminal bacteria colonizing CtP generated 1,261,552 raw reads. After screening, 1,153,139 high-quality sequences were obtained, with an average read length of 413 bases. Good’s coverage for each group was higher than 99%, indicating that the identified sequences represented the majority of bacteria. Rarefaction curves of all samples are presented in Fig. 1A. It was evident that all curves approached a plateau, indicating that the sequencing depth was sufficient and adding more operational taxonomic units (OTU) results in no change in the slope of the curve. High-quality reads were clustered into 2,752 microbial OTU at 3% divergence, among which 726 OTU were found in all groups and accounted for 26.38% of the total OTU, indicating the presence of a typical microbiome (Fig. 1B). The groups of 1 to 48 h had 26, 82, 50, 92, 92, 54, and 73 exclusive OTU, respectively.

Ace and Chao indexes are used to evaluate the abundance of the microbial community, while the Shannon and Simpson indexes are commonly used to measure the diversity of the microbial community (28). For bacterial Alpha diversity, observed OTU, Ace, Chao, and Shannon indexes first increased from 1 h to 24 h, then decreased from 24 h to 48 h (Table 4). The results suggested that a highly diverse rumen bacterial community colonized CtP rapidly and continuously changed during a 48-h incubation period. Rumen microbes differ in their functionality and their capability to use plant resources. A higher diversity index indicated that the microbial community's metabolic functional diversity was more extensive (29). Microbiota with a high diversity and richness are considered more stable and can use resources more efficiently (30). The principal coordinate analysis (PCoA) based on the unweighted and weighted UniFrac distance was used to characterize the Beta-diversity of rumen bacterial community (Fig. 1C and D). Samples at 8 h, 12 h, and 24 h were separated from samples of 1 h, 2 h, 4 h, and 48 h in the PCoA plot. The analysis of similarity (ANOSIM) revealed a significant difference in bacterial community attached to CtP among different time points (*P* < 0.05).

**TABLE 4:** Changes of the Alpha diversity of bacteria and archaea attached to citrus pomace within a 48-h *in situ* incubation in the rumen

Item	Time							SEM	*P* value
	1 h	2 h	4 h	8 h	12 h	24 h	48 h		
Bacteria									
Observed OTU	825^*b*^	822^*b*^	1039^*ab*^	1372^*a*^	1372^*a*^	1446^*a*^	1038^*ab*^	64.0	0.013
Ace	980^*b*^	973^*b*^	1229^*b*^	1590^*ab*^	1572^*ab*^	1655^*a*^	1192^*ab*^	73.8	0.019
Chao	988^*b*^	974^*b*^	1239^*ab*^	1614^*ab*^	1590^*ab*^	1659^*a*^	1187^*ab*^	75.4	0.016
Shannon	4.97^*ab*^	4.46^*b*^	4.68^*b*^	5.76^*a*^	5.55^*a*^	5.81^*a*^	5.12^*ab*^	0.564	0.017
Simpson	0.018	0.042	0.041	0.008	0.015	0.009	0.018	0.0034	0.078
Archaea									
Observed OTU	39	35	30	15	29	18	33	2.5	0.051
Ace	41	40	32	17	34	29	35	2.4	0.138
Chao	40	37	30	16	31	22	35	2.4	0.068
Shannon	1.46^*ab*^	1.49^*a*^	1.19^*ab*^	1.20^*ab*^	1.21^*ab*^	1.10^*b*^	1.39^*ab*^	0.040	0.025
Simpson	0.314	0.329	0.395	0.380	0.388	0.411	0.355	0.0114	0.077

a–b Means within a row followed by different lowercase letters differ significantly from each other (*P* < 0.05).

**FIG 2:**
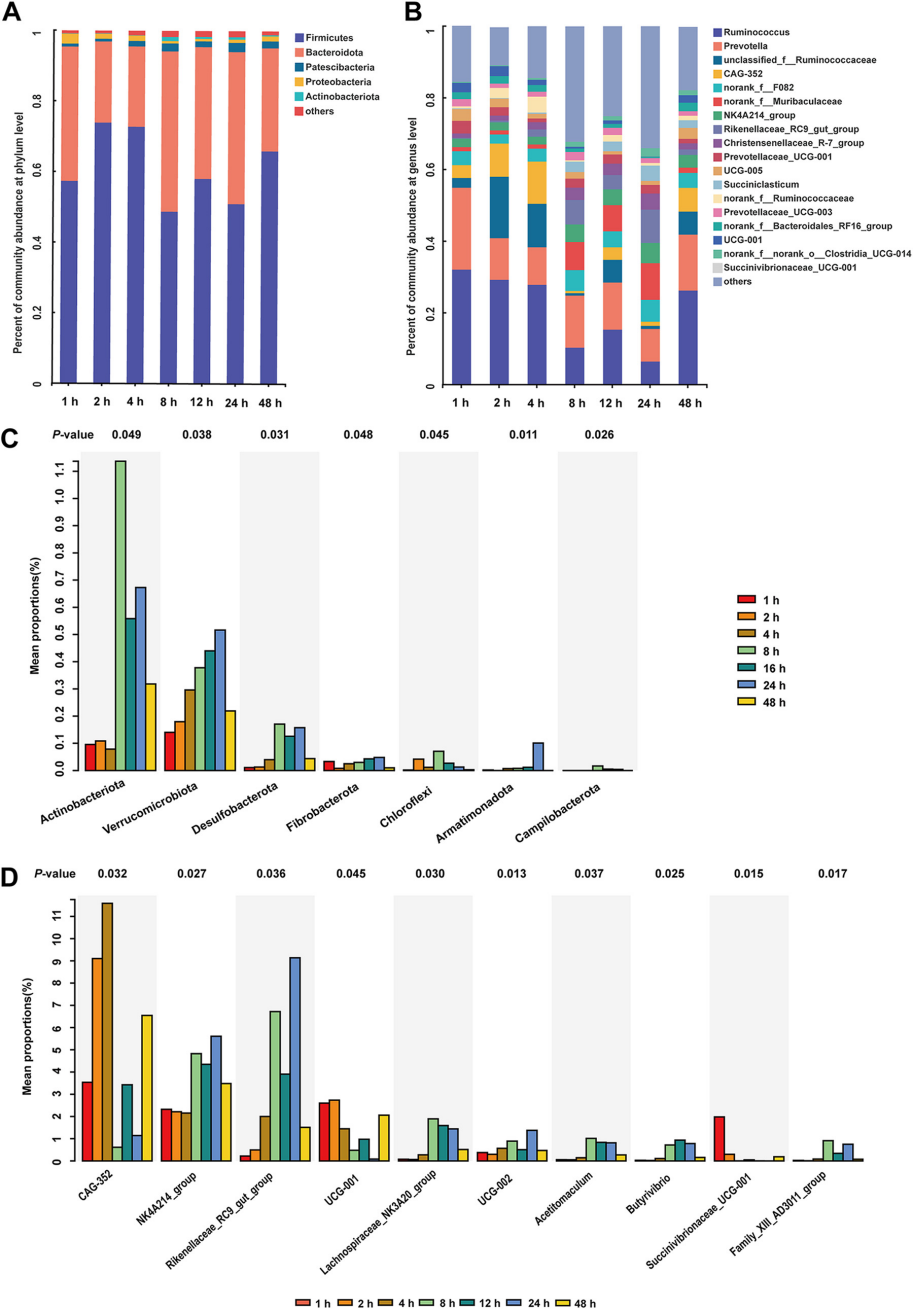
Dynamic changes of the composition of ruminal bacteria colonizing citrus pomace. (A) Relative abundances of bacterial dynamics at phylum level; (B) Relative abundances of bacterial dynamics at genus level; (C) Differential bacterial taxa at phylum level; (D) Differential bacterial taxa at genus level.

The microbial communities attached to CtP were affiliated with 28 bacterial phyla. Fig. 2A indicates the average relative abundances of the main bacterial phylum. Firmicutes (average 61.15%) and Bacteroidota (average 34.12%) were the most abundant phyla, which accounted for an average of 95.3% of the community (Fig. 2A). Major bacterial genera colonizing CtP within 48 h are shown in Fig. 2B, among which 19 genera (with relative abundance > 1%) were considered abundant core genera. *Ruminococcus* (15.07%) was the most abundant genus from the phylum Firmicutes, followed by unclassified_f__*Ruminococcaceae* (6.63%), *CAG-352* (5.21%), and *NK4A214*_*group* (3.62%), whereas *Prevotella* (13.90%) was the most abundant genus from the phylum Bacteroidota, followed by norank_f*__F082* (4.37%), norank_f__*Muribaculaceae* (4.32%), and *Rikenellaceae_RC9*_*gut*_*group* (3.48%). Colonization by ruminal microbes and available biomass are the cornerstones of rumen fermentation activities (31). The stacked column plot indicated that a highly diverse rumen microbiota colonized CtP quickly and persistently changed within the 48-h incubation period.

In the present study, we did not determine the bacterial community attached to the CtP at 0 h. Previous studies indicated that the Proteobacteria, which dominated on the surface of the forages at 0 h, were replaced by Firmicutes and Bacteroidetes at the first shift (29, 32, 33). In the present study, the bacterial communities attached to CtP at 1 h also consisted mainly of Firmicutes and Bacteroidetes. Anaerobic species belonging to both phyla Firmicutes and Bacteroidetes are known to degrade plant cell-wall polysaccharides (34). Although the temporal fluctuations of phyla Firmicutes and Bacteroidetes were observed across seven-time points, these two were the dominant phyla attached to CtP samples. The result is in accordance with data reported for rice straw, wheat straw, and alfalfa hay incubated *in situ* in the cow rumen (29, 35). Therefore, these phyla could play important roles in maintaining the efficiency of VFA production during CtP fermentation. In addition, *Prevotella* was reported to have an association with the degradation of oligosaccharides and hemicellulose (36). *Ruminococcus* is well recognized for its high fiber-degrading ability (37, 38), and this genus was reported to produce the most significant proportion of cellulases in the rumen, as well as a large amounts of hemicellulases and oligosaccharide-degrading enzymes (36). The present study revealed that the genera *Ruminococcus* and *Prevotella* were dominant in the bacterial community attached to CtP, indicating that these taxa played an important role in degrading fiber and oligosaccharides.

As shown in Fig. 2C, the relative abundances of Actinobacteriota, Verrucomicrobiota, Desulfobacterota, Fibrobacterota, Chloroflexi, Armatimonadota, and Campilobacterota increased first and then decreased during the 48-h fermentation period (*P* < 0.05). These differences in the microbial community across different time points are likely associated with drastic changes in the chemical composition of CtP. However, understanding of the ecology and biology of Actinobacteriota and Verrucomicrobiota in the rumen is minimal. Further studies to characterize the functions of these bacteria within Actinobacteriota and Verrucomicrobiota in the rumen are needed to evaluate whether these two taxa are linked to CtP fermentation. Fibrobacter succinogenes is the only known species from phylum Fibrobacterota, and this species is major cellulolytic bacteria in the rumen and produces succinate, the propionate precursor (39). In the present study, the dynamic characterization of Fibrobacterota attached to CtP biomass reflects, to a great extent, the changes in cellulose-degrading activity. Bacterial species belonging to Desulfobacterota could utilize acetate as one of the primary substrates to produce sulfide hydrogen in the rumen (40). The present study proposed that the significantly changed Desulfobacterota attached to CtP might be mainly due to the increased interaction between Desulfobacterota and acetogenic bacteria.

Of the dominant bacterial taxa colonizing CtP identified in the current research, most of the microbes are commonly stable. Linear discriminant analysis (LDA) effect size (LEfSe) was used to combine rank sum tests and taxonomic information to identify the biomarker taxa (logarithmic LDA score > 3.0) with the greatest impact on the structure of the community. The LDA histogram (Fig. S1) showed that there were 66 biomarkers, including one at 1 h, three at 2 h, one at 4 h, 19 at 8 h, 7 at 12 h, 33 at 24 h, and two at 48 h. Additionally, the relative abundances of genera *CAG-352*, *NK4A214_group*, *Rikenellaceae_RC9_gut*_*group*, *UCG-001*, *Lachnospiraceae_NK3A20*_*group*, *UCG*-*002*, *Acetitomaculum*, *Butyrivibrio*, *Succinivibrionaceae_UCG-001*, and *Family_XIII_AD3011_group* were significantly changed (*P* < 0.05) over time (Fig. 2D). *NK4A214_group* belongs to Oscillospiraceae family, which can degrade plant complex carbohydrates (41). Thus, CtP served as a carbon source for bacteria species of *NK4A214_group* colonization and growth. Some bacteria, such as *CAG-352*, *UCG-001*, and *UCG*-*002*, are of unknown function in the rumen. These genera belong to the Ruminococcaceae family, which is known to play an essential role in degrading plant fiber in the rumen (42). The genera *Lachnospiraceae_NK3A20*_*group* and *Butyrivibrio* belong family Lachnospiraceae, and the species within the family are the main butyrate-producing bacteria in the rumen (43). *Rikenellaceae_RC9_gut_group* in the family of Rikenellaceae, which is associated with either the primary or secondary degradation of structural carbohydrates (44). In short, the members of Ruminococcaceae, Lachnospiraceae, and Rikenellaceae played vital roles in colonizing and decomposing CtP biomass.

### Taxonomic profiling of ruminal archaea community attached to CtP.

**FIG 3:**
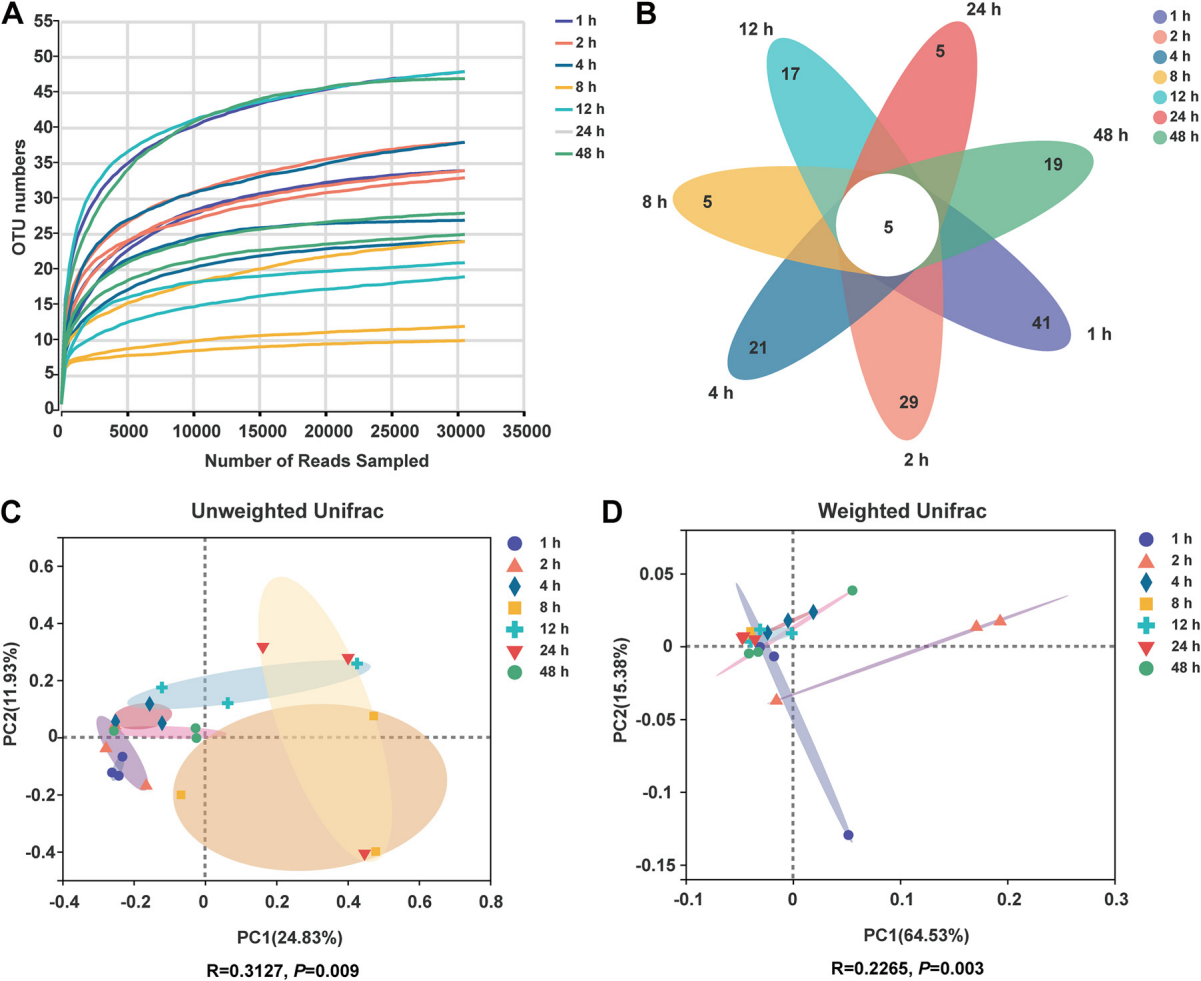
Dynamic changes of the structure of ruminal archaea colonizing citrus pomace. (A) The rarefaction curves; (B) Venn diagram at OTU level; (C) PCoA plot based on unweighted Unifrac distance; (D) PCoA plot based on weighted Unifrac distance.

As shown in Fig. 3A, rarefaction curves tend to reach a plateau, indicating that the sequencing depth was sufficient for further analysis. A Venn diagram showed that 41, 29, 21, 5, 17, 5, and 19 specific OTU existed in the 1, 2, 4, 8, 12, 24, and 48 h, respectively, with 5 OTU shared (Fig. 3B). From 2 to 24 h, the Shannon index increased, while after 24 h, the value decreased (Table 4). Other Alpha-diversity indices, including observed OTU, Ace, Chao, and Simpson, were not changed over time. The PCoA based on unweighted Unifrac distance showed apparent separation of different fermentation stages on the plot (Fig. 3B). ANOSIM confirmed a marked difference among groups (*P* < 0.05). These results suggested that CtP was also rapidly colonized by archaea, and the community structure of archaea attached to CtP changes over time.

**FIG 4:**
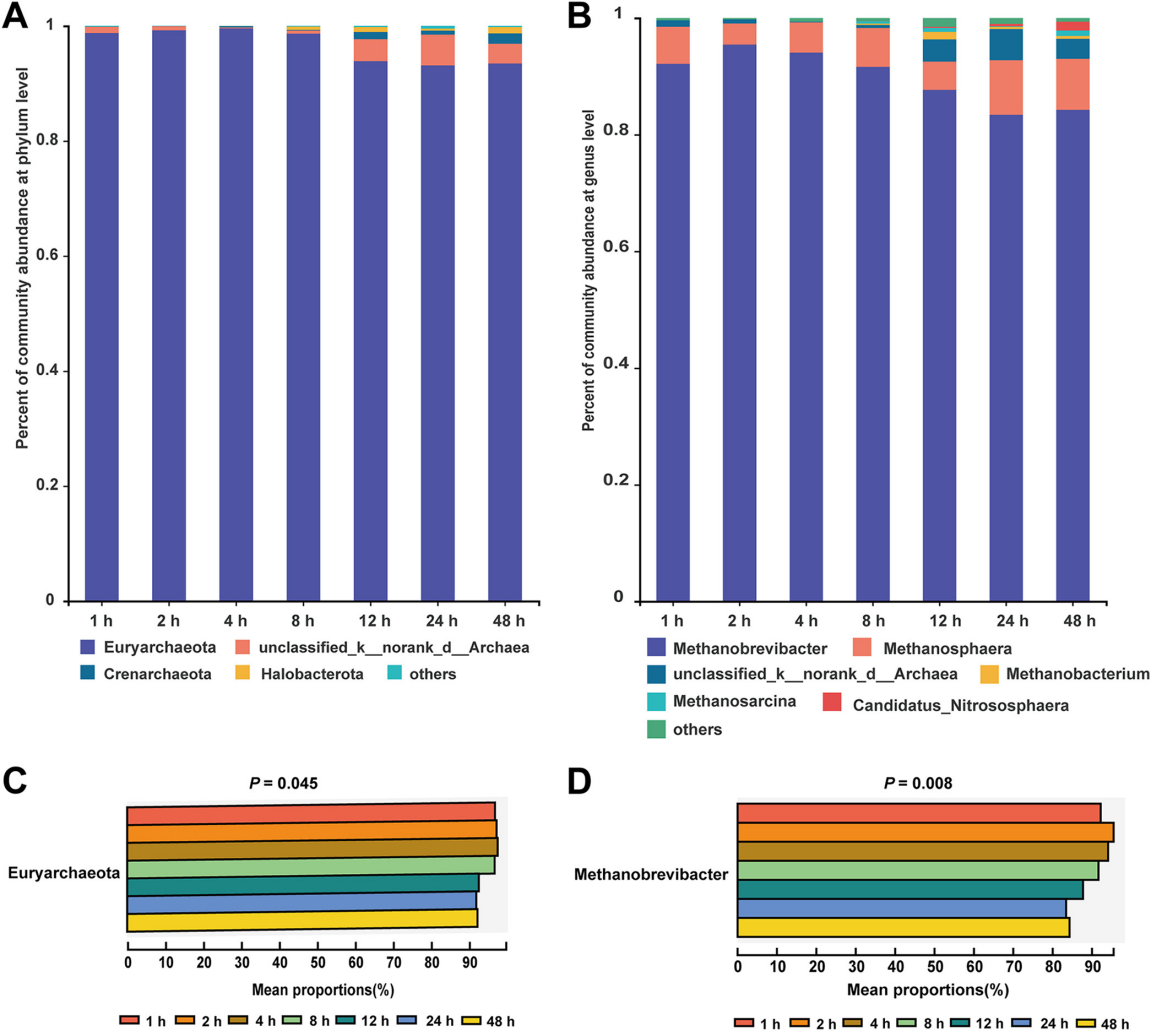
Dynamic changes of the composition of ruminal archaea colonizing citrus pomace. (A) Relative abundances of archaea dynamics at phylum level; (B) Relative abundances of archaea dynamics at genus level; (C) Differential archaea taxa at phylum level; (D) Differential archaea taxa at genus level.

The LDA histogram (Fig. S2) showed that there were 17 biomarker taxa within archaea, including one at 2 h, four at 4 h, 10 at 12 h, and two at 48 h. Changes in archaea community composition during the 48-h fermentation period at phylum and genus levels are presented in Fig. 4A and B. Taxonomic profiling showed that phylum Euryarchaeota and genus *Methanobrevibacter* accounted for most of the attached archaea. Regarding the relative abundance of archaea species, Euryarchaeota and *Methanobrevibacter* decreased significantly from 0.5 h to 48 h. However, other main archaea, such as *Methanosphaera* and *Methanobacterium* remained similar at different times. Bacteria and methanogens live in symbiotic relationship in the animal gut, and methanogen uses hydrogen produced by cellulolytic bacteria as electron donors to produce methane (45). These archaea members within *Methanobrevibacter* and *Methanosphaera* are predominant in ruminal methanogenesis (45). It has been documented that Euryarchaeota and *Methanobrevibacter* positively correlate with ruminal methane emissions (46). These reductions could be attributed to the inhibition effect of phytochemicals of CtP, such as flavonoids. However, the liquid-phase archaea and methane concentration in the rumen were not measured in the present study. Hence, further work is warranted to investigate methane production during CtP fermentation.

### Network analysis of microbial community.

**FIG 5:**
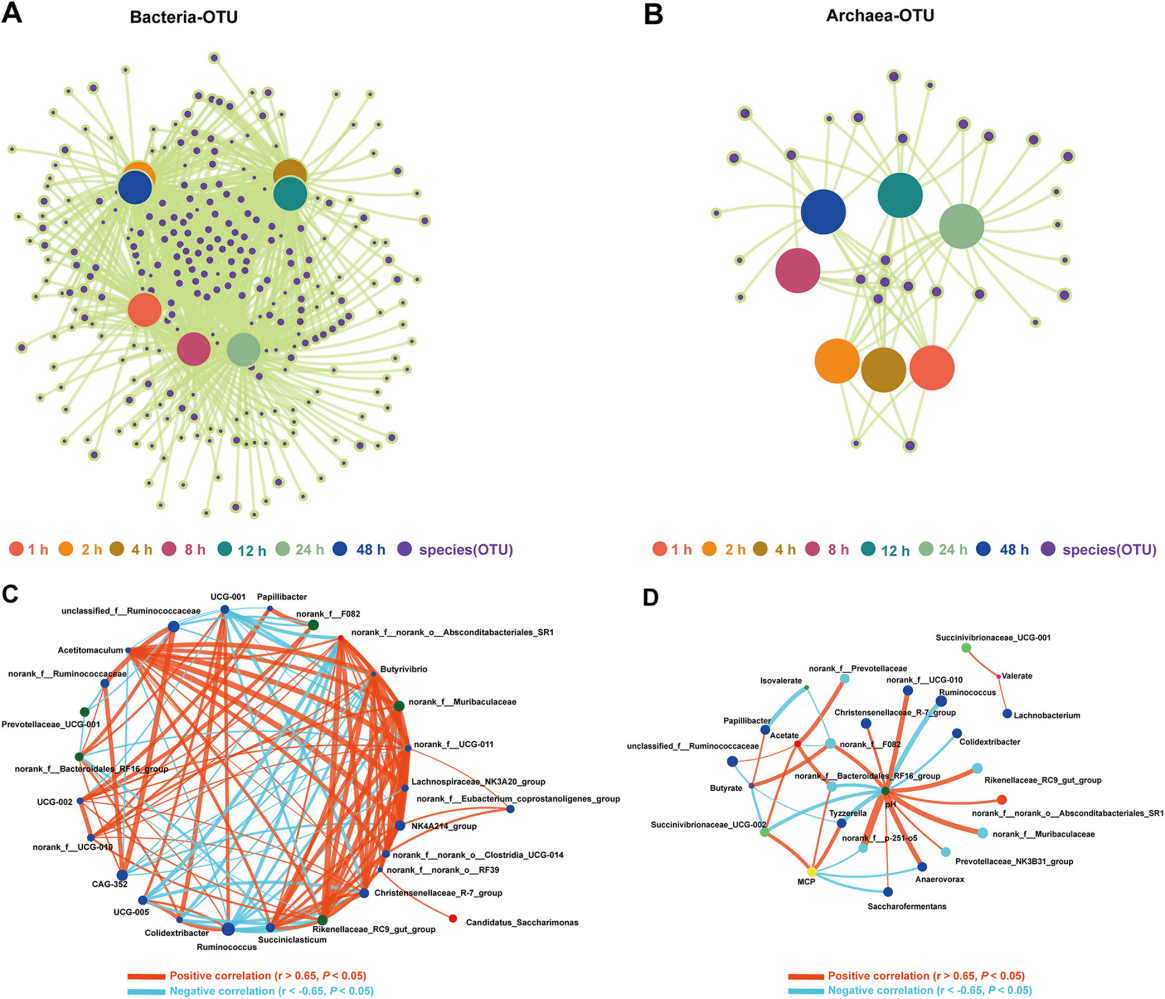
Network analysis of ruminal microbial taxa colonizing citrus pomace. (A) Cooccurrence network of bacterial community at OTU level; (B) Cooccurrence network of archaea community at OTU level; (C) Spearman’s correlation between rumen bacterial taxa in top 30 genera; (D) Spearman’s correlation between ruminal fermentation parameters and rumen bacterial taxa in top 50 genera. Only significant (*P* < 0.05; |r|>0.65) relationships are shown. The node size is proportional to the mean abundance.

The cooccurrence correlations among microbes are believed to play a role in determining community structure (47). As shown in Fig. 5A and B, the cooccurrence networks suggested significantly different topology structures for 1, 2, 4, 8, 12, 24, and 48 h. Additionally, Fig. 5C and Fig. S3 depicts the correlation network of the bacteria community (top 30 genera) and archaea community (top 20 genera). Bacterial networks were more complex than that archaea networks. The values of sample nodes’ connected degree in bacterial networks varied from 1,166 (1 h) to 2,037 (8 h), indicating a shift toward a more complex microbial network after the beginning of CtP fermentation. The rumen is a highly dynamic environment (48), and the microbiota can adapt and respond immediately to CtP biomass degradation. The bacterial correlation network consisted of 27 nodes (genera) and 276 edges, and significant positive and negative correlations accounted for 68.5% and 31.5% of bacterial networks. Most positive connections existed among genera taxa of phylum Firmicutes, such as *Acetitomaculum*, *Rikenellaceae_RC9_gut_group*, *Christensenellaceae_R-7_group*, and *Butyrivibrio*, whereas most negative connections from the genera taxa *UCG-001*, *Colidextribacter*, and *Ruminococcus*. Generally, positive correlations represent cross-feeding and niche overlap, but negative correlations are attributed to competition in the network (49). These findings suggested that members of Firmicutes play a significant role in regulating the relative abundances of various microbiota in colonizing and degrading CtP.

In order to better understand the relationship between fermentation parameters and kinetics of the top 30 bacterial genera during CtP fermentation, the Spearman correlation analysis was performed. As shown in Fig. 5D, pH was positively correlated with *Anaerovorax*, *Rikenellaceae_RC9_gut_group*, and negatively correlated with *Ruminococcus*. Acetate showed a positive correlation with *Succinivibrionaceae_UCG-002*. Butyrate was positively correlated with *Papillibacter*, and negatively correlated with *Succinivibrionaceae_UCG-002*. Isovalerate showed a negative correlation with *Papillibacter*. Valerate was positively correlated with *uccinivibrionaceae_UCG-001* and *Lachnobacterium*. In short, pH, acetate, butyrate, isovalerate, and valerate were closely related to the attached ruminal bacteria. A reduction in ruminal pH is generally associated with VFA accumulation (50). Most ruminal cellulolytic bacteria are pH-sensitive (51), and the accumulation of VFA is the limiting factor in microbial fermentation, especially in batch fermentation (8). However, VFA *in vivo* were continuously absorbed by the rumen wall of the host, and VFA accumulation is not present in the present study. Therefore, separating VFA might be feasible to maintain efficient VFA production *in vitro*. A membrane bioreactor can be used to separate VFA during long-term rumen fluid fermentation to maintain the high activity of rumen microorganisms (52).

### Conclusions.

The diverse microbes in the rumen and their symbiotic interactions make them a treasure trove for the sustainable production of value-added products from food waste. This study analyzed the dynamic changes in CtP fermentation performance and attached microbial communities in the rumen. CtP becomes rapidly colonized by microorganisms from the rumen. Chemical analyses indicated that decreased cellulose biomass of CtP was concomitant with increased VFA production in the rumen. The microbial analysis showed that genera *Ruminococcus*, *Prevotella*, *CAG-352*, *NK4A214_group*, *Rikenellaceae_RC9_gut_group*, and *Methanobrevibacter* would dominate over the fermentation period. In addition, the relative abundances of phyla Actinobacteriota, Verrucomicrobiota, Desulfobacterota, Fibrobacterota, and Euryarchaeota were significantly different among various time points. These findings may provide a deep understanding of accelerating the exploitation of citrus waste in anaerobic digestion with fresh rumen fluid for VFA production by constructing, manipulating, and enriching rumen microbial consortia. In the future, the development of multiomics, bioinformatics, and microbial pure culture techniques will be helpful to explore the functions and interrelations of rumen microorganisms dominating CtP digestion, and reveal the complex process of hydrolysis, acidogenesis and methanogenesis of citrus waste.

## MATERIALS AND METHODS

### Experimental design.

The animal procedures and uses were approved by the Animal Care Committee of Beijing University of Agriculture (protocol no. BUA2021146; Beijing, China). Three mature nonlactating Chinese Holstein cows (average body weight 540 ± 11 kg) with permanent rumen fistulas were used as experimental animals for *in situ* incubation. Cows were housed in a tie-stall barn and had free access to water. All cows were fed the same total mixed ration *ad libitum* once daily. The diet contained (DM basis): 31.7% of alfalfa silage, 21.2% of corn silage, 24.3% Chinese wildrye, 8.2% ground corn grain, 6.6% soybean meal, 5.3% of soybean hull, 0.7% of calcium carbonate, 0.5% of calcium orthophosphate, 0.5% of sodium chloride, and 1% of mineral and vitamin premix. Cows were individually housed in 3 stalls in a barn. The animals had received the ration more than 1 month to equilibrate the ruminal populations before the experiment was conducted. During the trial, the management of cows remained unchanged.

The CtP was obtained from Shaanxi Xiazhou Biotechnology (Xi’an, China). Samples of CtP were oven-dried at 65°C for 48 h, and ground through a 2-mm sieve in a Wiley mill (Thomas Scientific, Swedesboro, NJ). The chemical composition of CFE is presented in Table S1. One hundred grams of CtP were enclosed in a nylon bag (10 × 20 cm; pore size = 50 μm). The nylon bag (a total of 21 bags per cow, 3 bags for each time point) was placed in the rumen of dairy cows prior to morning feeding. For the determination of fermentation parameters, rumen digesta samples were collected at 1, 2, 4, 8, 12, 24, and 48 h through cannula from multiple sites (i.e., anterior dorsal, anterior ventral, medium ventral, posterior dorsal, and posterior ventral) within the rumen of dairy cows. Rumen content was strained through 4 layers of cheesecloth, and the pH was measured immediately with a digital pH meter (PHS-3C; Shanghai Yueping Scientific Instrument Co., Ltd., Shanghai, China). All rumen fluid samples were subsampled (10 mL) and stored at −80°C until analysis. For the analysis of cellulase activity and microorganisms colonizing CtP, the nylon bag was removed after incubating 1, 2, 4, 8, 12, 24, and 48 h, then rinsed using sterile saline to remove transient and loosely attached microbes. The samples were stored at −80°C until DNA extraction.

### Rumen fermentation characteristics.

The VFA concentrations of rumen fluid samples were analyzed using a gas chromatograph (GC-7890B; Agilent Technologies, Santa Clara, CA) equipped with a capillary column (30 m × 0.25 mm × 0.25 μm; DB-FFAP; Agilent Technologies), and a flame ionization detector. The oven temperature was 170°C held for 4 min and was raised to 185°C at a rate of 5°C/min, followed by 3°C/min to 240°C, maintaining this temperature for 1 min. The injector and detector temperatures were maintained at 250°C and 300°C, respectively. For VFA analysis, 4 mL of strained rumen fluid was mixed with 1 mL of 25% metaphosphoric acid. The rumen extract was centrifuged at 20,000 × *g*, and the supernatant was recovered and stored at −20°C until GC analysis. The samples were dosed by an autosampler at an injection size of 1 μL using the split method and a 25:1 splitting ratio. Nitrogen was used as the carrier gas. Ruminal ammonia nitrogen was determined according to the method of Broderick and Kang (53) on a microplate reader (Multiskan FC, Thermo Fisher, NY). Ruminal microbial crude protein was analyzed according to the description of Makkar et al. (54).

**Microbial cellulose enzyme activity.** One gram of CtP residue in the nylon bag was placed into a centrifuge tube. Twenty milliliters of sodium phosphate buffer solution (0.01 mol/L, pH 6.8) and 2.5 mL carbon tetrachloride were added to the sample. The centrifuge tube was incubated at 37°C with constant shaking, and then the enzyme-containing supernatant was obtained by centrifuging at 29,000 × *g* for 15 min at 4°C. Pectinase, carboxymethyl cellulase, and xylanase activity were determined with pectin, sodium carboxymethyl cellulose, and xylan (Beijing Solarbio Science & Technology Co., Ltd., Beijing, China) as the substrates according to Agarwal et al. (55).

**Biomass degradation.** The DM (method 930.15), crude protein (method 973.48), ash (method 942.05), and ether extract (method 920.39) of CtP were determined according to AOAC (56). The NDF and ADF were analyzed sequentially using an Ankom^200^ fiber analyzer (ANKOM Technology Corp., Macedon, NY) with ANKOM filter bag technique. Relative biomass degradation of DM, NDF, and ADF for different time points was calculated, and the CtP sample at 0 h was used as the reference with biomass at 100%.

### Relative abundance of specific rumen microbes by real-time qPCR.

The DNA was extracted with DNA kit (E.Z.N.A. Soil DNA kit, Omega Biotek, Norcross, GA) according to the manufacturer’s recommendations. The purity and concentration of extracted DNA were evaluated by a NanoDrop 1000 spectrophotometer (NanoDrop Technologies, Rockland, DE) and 1% agarose gel electrophoresis. The target microbes were Methanogens, *Anaerovibrio lipolytica*, Butyrivibrio fibrisolvens, Fibrobacter succinogenes, *Megaspheara elsdenii*, Prevotella brevis, *Rumicoccus albus*, *Rumicoccus flavefaciens*, and Streptococcus bovis. Primer sequences of these targeted microbes are listed in Table S2. A total of 2 μL of DNA template (10 ng/μL) was added to the reaction system containing 0.8 μL of each primer (10 μmol/L), 10 μL of SYBR green (Beijing Solarbio Science & Technology Co., Ltd., Beijing, China), and DNA/RNA free water adjusting to the total volume (20 μL) in triplicate for each sample. Reactions were conducted in a Roche LightCycler96 system (Roche Diagnostics Deutschland GmbH, Mannheim, Germany) with the following procedure: 1 min at 95°C, 40 cycles of 15 s at 95°C and 30 s at 60°C (or relevant based on primers *T_m_*), and 68°C for 1 min for the extension. The cycle threshold (Ct) was used to calculate the fold change of each rumen microbe relative to the total bacteria. The relative abundance of theses target microbes was calculated by the following equation: $\text{relative abundance }={\text{ 2}}^{-[\text{Ct}\left(\text{target}\right)-\text{Ct}(\text{total bacteria})]}$. Subsequently, the data were log-2 transformed prior to statistical analysis.

### Bacterial and archaea community analysis.

The bacterial and archaea communities of rumen-incubated CtP samples (three biological replicates per time point) were analyzed by sequencing the 16S rRNA gene via an Illumina MiSeq platform. For bacteria, the primers 338F (5′-barcode-ACTCCTRCGGGAGGCAGCAG-3′) and 806R (5′-GGACTACCVGGGTATCTAAT-3′) with a unique eight-base “barcode” sequence were used to for the amplification of 16S rRNA from V3-V4 region. For archaea, the primers 524F10extF (5′-TGYCAGCCGCCGCGGTAA-3′) and Arch958RmodR (5′-YCCGGCGTTGAVTCCAATT-3′) were used to amplify the V4-V5 region of 16S rRNA gene. PCR amplification was performed in triplicate with a 25-μL reaction mixture consisting of 2 μL DNA template, 12.5 μL 2×*Taq* PCR MasterMix, 2.5 μL of each primer, and ddH_2_O to adjust the final volume. The PCR products were assessed by 2% agarose gel electrophoresis and purified using a QIAquick Gel Extraction kit (Qiagen, Hilden, Germany). Amplicons were then pooled in equimolar proportions to generate amplicon libraries. Amplicon sequencing (2 × 300 bp) was conducted at an Illumina MiSeq sequencing system (Illumina, San Diego, CA).

Paired-end reads were merged with FLASH (Version 1.2.11). Sequence reads were processed and analyzed using the Quantitative Insights into Microbial Ecology (QIIME) pipeline software (Version 1.9.1). Average linkage clustering was used to assign reads to OTU based on a 97% similarity. OTU raw read counts were normalized against the total number of quality-filtered reads to calculate relative abundances. Taxonomic assignments for each OTU were conducted using the RDP classifier (Version 2.13) against the SLIVA 16S rRNA database (Version 138). Alpha-diversity indices of ACE, Chao1, Shannon, and Simpson were calculated using Mothur (Version 1.30.2). Beta diversity was analyzed with PCoA based on weighted and unweighted UniFrac distances using QIIME. The ANOSIM statistical test was conducted to identify the statistical significance in Beta-diversity among groups.

### Statistical analysis.

Statistically significant differences in physicochemical data, including rumen fermentation parameters, microbial cellulose enzyme activity, biomass loss, and real-time qPCR data, were analyzed using SAS 9.4 (SAS Institute Inc.) with one-way ANOVA and the general linear model procedure. All values were reported as least-squares mean. Significance was considered at *P *≤* *0.05. The significant difference between means was investigated using Duncan’s multiple range test. The Kruskal-Wallis test with the false discovery rate correction (FDR) was used to assess the changes in the relative abundance of microbial taxa at different time points, and FDR-corrected *P* values below 0.05 were considered significant. Spearman’s rank correlations were used to evaluate the relationship between bacterial genera and rumen fermentation parameters.

### Data availability.

The raw sequences obtained have been deposited in NCBI Sequence Read Archive under the accession number PRJNA832488.
