# Supplementary material for: Characterization of the Dynamic Changes of Ruminal Microbiota Colonizing Citrus Pomace Waste during Rumen Incubation for Volatile Fatty Acid Production
Source: Microbiol Spectr. 2023 Mar 2;11(2):e03517-22. doi: 10.1128/spectrum.03517-22 (PMC10101060; doi:10.1128/spectrum.03517-22)
Supplement: Supplemental file 1 — Supplemental material. Download spectrum.03517-22-s0001.pdf, PDF file, 0.5 MB [file spectrum.03517-22-s0001.pdf]

Supplementary Materials

Table S1 Chemical composition of citrus pomace waste.

Item	Contents, % of dry matter
Dry matter	87.67
Organic matter	93.78
Ash	6.22
Neutral detergent fiber	32.15
Acid detergent fiber	16.36
Lignin	9.19
Crude protein	8.93
Ether extract	3.42
Soluble polysaccharide	9.87
Total polyphenol	7.17
Total flavonoids	4.04

Table S2 Species-specific primers for the quantification of selected rumen microbes using a real-time qPCR assay.

Target bacterial species		Primer sequence (5'- 3')	Tm (°C)	Reference
Total bacteria	F	CGGCAACGAGCGCAACCC	60	Denman et al. (2006)
	R	CCATTGTAGCACGTGTGTAGCC		
Methanogens	F	TTCGGTGGATCDCARAGRGC	58	Denman et al. (2007)
	R	GBARGTCGWAWCCGTAGAATCC		
Anaerovibrio lipolytica	F	GAAATGGATTCTAGTGGCAAACG	60	Abdelmegeid et al. (2018)
	R	ACATCGGTCATGCGACCAA		
Butyrivibrio fibrisolvens	F	ACACACCGCCCGTACCA	65	Klieve et al. (2001)
	R	TCCTTACGGTTGGGTCACAGA		
Fibrobacter succinogenes	F	GCGGGTAGCAAACAGGATTAGA	60	Abdelmegeid et al. (2008)
	R	CCCCCGGACACCCAGTAT		
Megaspheara elsdenii	F	AGATGGGGACAACAGCTGGA	60	Abdelmegeid et al. (2008)
	R	CGAAAGCTCCGAAGAGCCT		
Prevotella brevis	F	GGTTTCCTTGAGTGTATTCGACGTC	64	Stevenson et al. (2007)
	R	CTTTCGCTTGGCCGCTG		
Ruminococcus albus	F	ACGTCRTCCMCACCTTCCTC	62	Koike et al. (2001)
	R	CCTCCTTGCGGTTAGAACA		
Ruminococcus flavefaciens	F	CGAACGGAGATAATTTGAGTTTACTTAGG	60	Denman et al. (2006)
	R	CGGTCTCTGTATGTTATGAGGTATTACC		
Streptococcus bovis	F	TTCCTAGAGATAGGAAGTTTCTTCGG	57	Abdelmegeid et al. (2008)
	R	ATGATGGCAACTAACAATAGGGGT		

Abdelmegeid MK, Elolimy AA, Zhou Z, Lopreiato V, McCann JC, Loor JJ. Rumen-protected methionine during the peripartal period in dairy cows and its effects on abundance of major species of ruminal bacteria. J Anim Sci Biotechnol. 2018; 9:17. https://doi.org/10.1186/s40104-018-0230-8.

Denman SE, Mcsweeney CS. Development of a real-time PCR assay for monitoring anaerobic fungal and cellulolytic bacterial populations within the rumen. Fems Microbiol Ecol. 2006; 58:572-82. https://doi.org/10.1111/j.1574-6941.2006.00190.x.

Denman SE, Tomkins NW, Mcsweeney CS. Quantitation and diversity analysis of ruminal methanogenic populations in response to the antimethanogenic compound bromochloromethane. Fems Microbiol Ecol. 2007; 62: 313-22. https://doi.org/10.1111/j.1574-6941.2007.00394.x.

Koike S, Kobayashi Y. Development and use of competitive PCR assays for the rumen cellulolytic bacteria: *Fibrobacter succinogenes*, *Ruminococus albus* and *Ruminococcus flavefaciens*. FEMS Microbiol Lett. 2001; 204:361-6. https://doi.org/10.1111/j.1574-6968.2001.tb10911.x.

Stevenson DM, Weimer PJ. Dominance of *Prevotella* and low abundance of classical ruminal bacterial species in the bovine rumen revealed by relative quantification real-time PCR. Appl Microbiol Biotechnol. 2007; 75:165-74. https://doi.org/10.1007/s00253-006-0802-y.

Sylvester JT, Karnati SKR, Yu Z, Mark M, Firkins JL. Development of an assay to quantify rumen ciliate protozoal biomass in cows using real-time PCR. J Nutr. 2004; 134:3378-84. https://doi.org/10.1093/jn/134.12.3378.

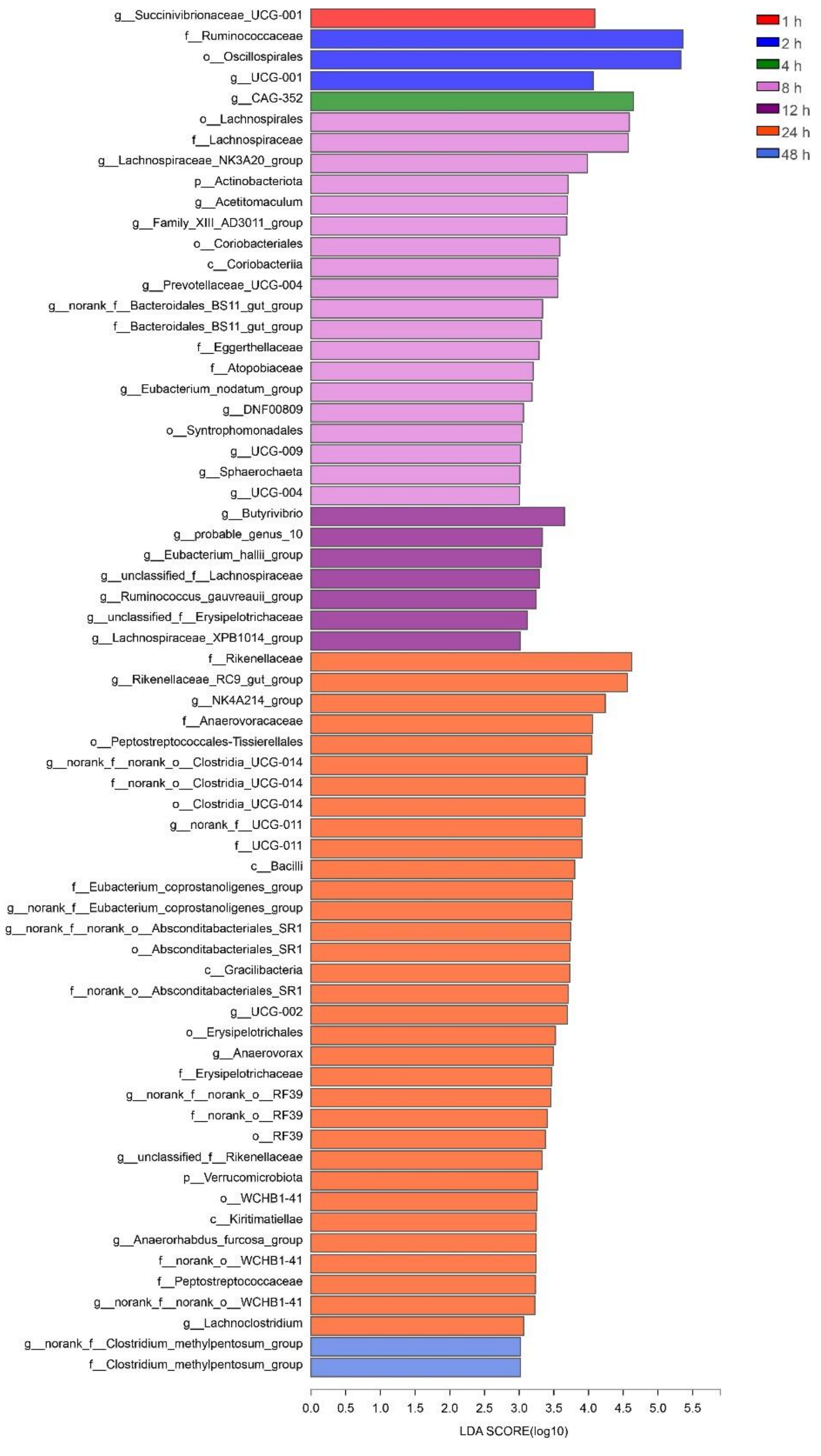

Fig. S1. Linear discriminant analysis (LDA) effect size (LEfSe) ($P < 0.05$ by Kruskal-Wallis test; $P < 0.05$ by Wilcoxon rank-sum test; logarithmic LDA score of>3.0) of the ruminal bacteria attached to citrus pomace. Taxonomic rank labels are provided before bacterial names; p_, c_, o_, f_, and g_indicate phylum, class, order, family, and genus, respectively. The greater the LDA score of the biomarker taxon mean value, the greater the influence of species abundance on the difference in the microbial community in the different time points.

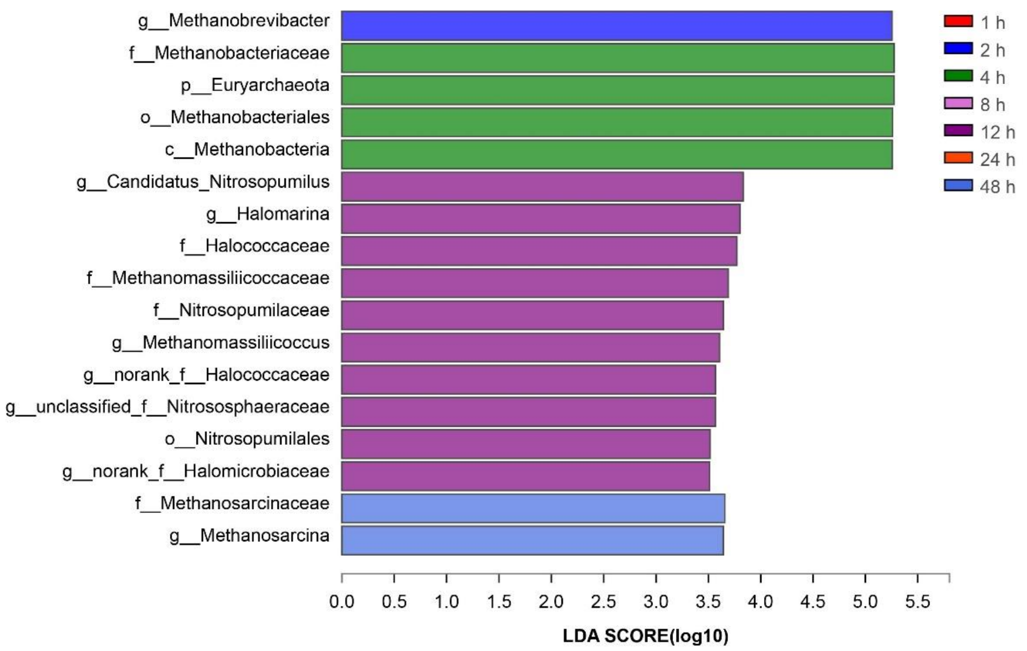

Fig. S2. Linear discriminant analysis (LDA) effect size (LEfSe) ($P < 0.05$ by Kruskal-Wallis test; $P < 0.05$ by Wilcoxon rank-sum test; logarithmic LDA score of>3.0) of the ruminal archaea attached to citrus pomace. Taxonomic rank labels are provided before bacterial names; p_, c_, o_, f_, and g_indicate phylum, class, order, family, and genus, respectively. The greater the LDA score of the biomarker taxon mean value, the greater the influence of species abundance on the difference in the microbial community in the different time points.

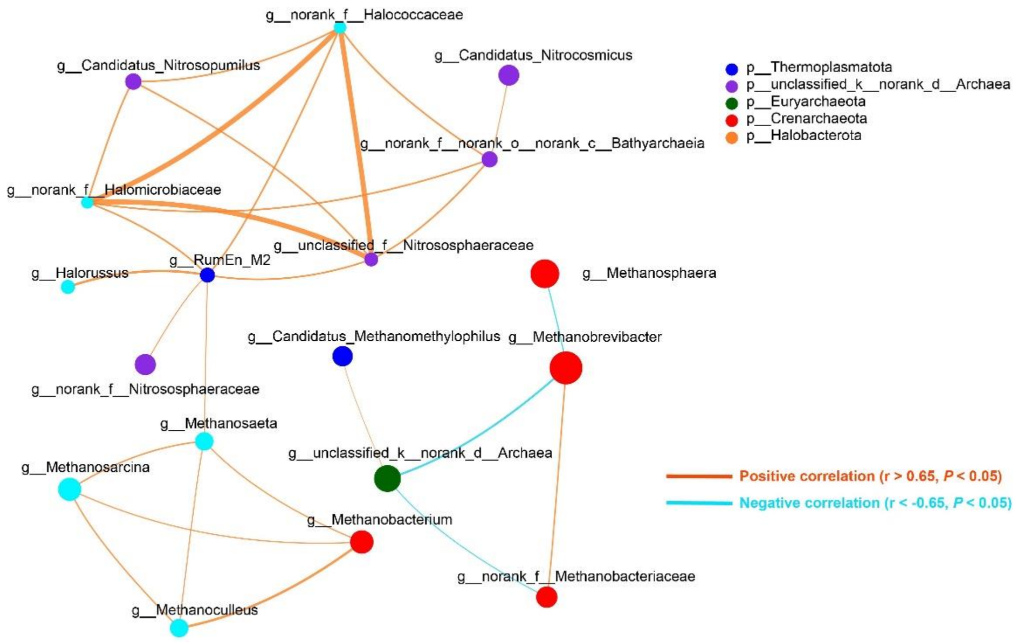

Fig. S3. Relationships between rumen archaea taxa at the genus level. Only significant ($P < 0.05$; $|r|>0.65$) relationships are shown. Orange edges indicate positive relationships, and green edges indicate negative relationships. The node size is proportional to the mean abundance.
